# Comparative analysis of high-speed videolaryngoscopy images and sound data simultaneously acquired from rigid and flexible laryngoscope: a pilot study

**DOI:** 10.1038/s41598-021-99948-9

**Published:** 2021-10-14

**Authors:** Wioletta Pietruszewska, Marcin Just, Joanna Morawska, Jakub Malinowski, Joanna Hoffman, Anna Racino, Magda Barańska, Magdalena Kowalczyk, Ewa Niebudek-Bogusz

**Affiliations:** 1 Department of Otolaryngology, Head and Neck Oncology, Medical University of Lodz, Lodz, Poland; 2 Diagnova Technologies, Wroclaw Technology Park, Wroclaw, Poland

**Keywords:** Medical research, Medical imaging, Physical examination

## Abstract

High-Speed Videoendoscopy (HSV) is becoming a robust tool for the assessment of vocal fold vibration in laboratory investigation and clinical practice. We describe the first successful application of flexible High Speed Videoendoscopy with innovative laser light source conducted in clinical settings. The acquired image and simultaneously recorded audio data are compared to the results obtained by means of a rigid endoscope. We demonstrated that the HSV recordings with fiber-optic laryngoscope have enabled obtaining consistently bright, color images suitable for parametrization of vocal fold oscillation similarly as in the case of the HSV data obtained from a rigid laryngoscope. The comparison of period and amplitude perturbation parameters calculated on the basis of image and audio data acquired from flexible and rigid HSV recording objectively confirm that flexible High-Speed Videoendoscopy is a more suitable method for examination of natural phonation. The HSV-based measures generated from this kymographic analysis are arguably a superior representation of the vocal fold vibrations than the acoustic analysis because their quantification is independent of the vocal tract influences. This experimental study has several implications for further research in the field of HSV application in clinical assessment of glottal pathologies nature and its effect on vocal folds vibrations.

## Introduction

**Figure 1:**
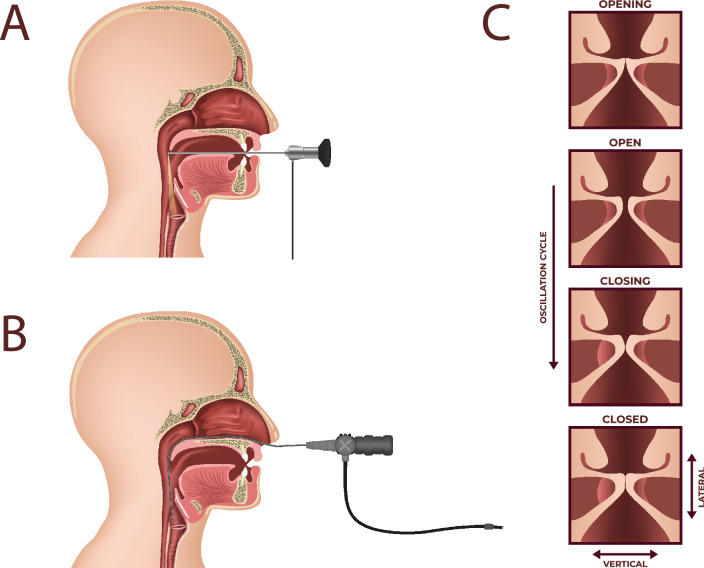
Schematic illustration: recording of the vocal fold vibration by means of High -Speed Videolaryngoscopy using (**A**) rigid endoscope, (**B**) flexible endoscope, (**C**) image of one vibration cycle of the vocal folds during phonation.

The human voice is a unique phenomenon and its production is based on three important functions of vocal tract: breathing, phonation and resonance. The superior function in the complex process of voicing is phonation determined by vibrations of vocal folds stretched horizontally inside the glottis—the medium part of the larynx referred to as voice organ. The value of direct visualization of laryngeal structures and vocal fold vibratory patterns for reaching an appropriate diagnosis and determining the best therapeutic approach has been frequently reported in literature^1–4^. Digital imaging techniques enable the exploration of novel visualisation modalities of the vocal folds during phonation^4–7^. High-speed videoendoscopy (HSV) of the larynx is a complementary tool to commonly used laryngovideostroboscopy (LVS). Both these tools enable the investigating of vocal fold vibratory function in normal and pathological states of glottis. The disadvantage of LVS is that a single slow motion cycle of the vocal folds is built from a montage of tens of true vibratory cycles of the vocal folds. Thus, valid results of the LVS imaging techniques can only be obtained if a sufficiently regular vibration periodicity of the vocal folds is sustained^8–10^. Consequently, a number of non-periodical voice disorders cannot be diagnosed with the use of LVS^11,12^. High Speed Videoendoscopy overcomes a number of problems associated with stroboscopy. High sampling rate with adequate spatial resolution allows for the observation of each cycle of vocal fold vibration^13–15^. The HSV recording technique enables the visualisation of the main within-glottic cycle vibratory phases, namely the opening, open, closing and closed phases (Fig. 1). This results in periodic interruption of the airflow traveling through the glottis that produces audible sound during phonation. High Speed Videoendoscopy examination enables the visualization of aperiodic and irregular vocal fold vibrations as well as phonatory gestures such as voice onset (voice initiation, beginning of phonatory phase) and offset (voice termination, end of phonatory phase)^16–20^.

A number of studies have presented HSV system as a platform particularly suitable for building the glottovibrogram, thus allowing for visualization of vocal folds kinematics and their quantitative assessment^21,22^. The glottovibrogram is a two-dimensional spatiotemporal representation of the glottal area changes during phonation. The computed geometric parameters of the glottal area in each single image of the video sequence can be quantified as the function of glottal area over time, i.e. Glottal Area Waveform (GAW)—a plot describing the increase and decrease of the glottal area during individual glottal cycles^23–26^. From the GAW one can calculate parameters characterising vocal fold vibrations in the form of parameters exclusively connected to the GAW like the Open Quotient (OQ), i.e. the duration of the open phase within the total glottal cycle, as well as parameters which can be determined both from the GAW and acoustic signals of voice such as fundamental frequency F0 and frequency perturbation parameters, e.g. jitter and amplitude perturbation parameters e.g. shimmer^27–30^.

HSV-based analysis measures are arguably a richer representation of the vocal fold vibration features than acoustic analysis parameters, because their quantification is independent of the vocal tract influences. Acoustic signals contain contributions from the glottal source and from the supraglottal structures of the vocal tract (e.g. pharynx, nose, paranasal sinuses). The problem associated with this feature has been described in literature^7,31,32^, nevertheless, the use of acoustic measures calculated on the basis of simultaneous recording of voice signals reveals useful information related to laryngeal pathophysiology^2,7,24,33,34^.

The majority of the available HSV systems rely on the high speed camera attached to rigid endoscopes^1^. The introduction of flexible endoscopes for transnasal laryngeal imaging has facilitated the progress in voice research and clinical practice, making it possible to observe more natural laryngeal function or to examine patients with overactive gag reflex. High-Speed Videoendoscopy with the use of a flexible fiberoptic nasolaryngoscope shows vocal fold vibrations in a physiological, undisturbed way (Fig. 1). It has been shown, however, in those limited studies that flexible HSV image still makes edge tracking for extraction of Glottal Area Waveform (GAW) and objective analysis difficult^35–37^. Despite many efforts undertaken, designing effective algorithms for making image enhancement in flexible HSV examination suitable for quantitative assessment of vocal fold vibrations still remains a challenging task.

The main objective of the study was to demonstrate that acquiring the larynx images recorded by means of transnasal fiberoptic High-Speed Videoendoscopy in clinical settings is possible. In order to prove this conjecture, the particular objectives of the study included the comparison of flexible HSV image data and image data obtained from a rigid HSV. This comparison was based on quantitative measurements describing vocal fold vibrations and verification of quantitative analysis of the HSV images by acoustic analysis of synchronously registered voice.

## Material and methods

The High Speed Laryngoscopic images were recorded in the Department of Otolaryngology, Head and Neck Oncology, Medical University of Lodz, Poland.

For the assessment of rigid and flexible HSV systems applicability, we examined a group of 15 subjects, and for detailed presentation two females were selected: a normophonic case (Subject 1) and an organic disorder case (Subject 2). Prior to High-Speed Videoendoscopy (HSV) examination, the Subjects of the study underwent a multidimensional assessment of vocal function according to the protocol put forward by the Committee on Phoniatrics of the European Laryngological Society (ELS) including: laryngovideostroboscopy (LVS), perceptual assessment (GRBAS), acoustic analysis of voice, self-assessment of voice (Voice Handicap Index; VHI) and aerodynamic assessment (Maximum Phonation Time; MPT). Vocal fold function was assessed by means of High Speed Videoendoscopy during repeated stable phonation of sustained vowel /i:/ at a comfortable pitch and loudness. Approval for this study was granted by the Ethical Committee of the Medical University of Lodz (decision no. RNN/96/20/KE 08/04/2020).

All procedures performed in studies involving human participants were in accordance with the ethical standards of the institutional and national research committee and with the 1964 Helsinki Declaration and its later amendments or comparable ethical standards.

Informed consent was obtained from all individual participants involved in the study.

### HSV instrumentation and postprocessing

First, HSV recordings were performed by means of rigid, oval-shaped endoscope Fiegert -Endotech ϕ12.4/7.2 with light input in Storz standard matched to 4.8 mm fiber optic light cable. Next, flexible fiber-naso-pharyngo-laryngoscope Fiegert-Endotech ϕ3.4 with external light input (matched to 3.5 mm fiber optic light cable) was used.

The HSV images of the larynx were registered with Advanced Larynx Imager.

System (ALIS) manufactured by Diagnova Technologies, equipped with innovative endoscope laser light source (ALIS Lum-MF1) and laryngeal high speed camera (ALIS Cam HS-1) with a CMOS image sensor with a colour filter array (the classic Bayer filter) and a global shutter (http://diagnova.eu/pages/offer/illu-minatorMF1.html, 2020-05-11). After the debayering process the spatial resolution of digital images for frame-rates ranging from 2000 to 3200 images per second range from 544 × 512 to 416 × 416 pixels correspondingly. In the high speed camera mode only the central region of the sensor is captured and the image covers only a small part of the entire field of view, thus the geometric distortions of the camera are negligible and were not corrected. Moreover, fixed pattern noise and white balance correction are implemented in the video acquisition software offered by the hardware manufacturer. The laser light source (Diagnova 2019) produced a narrowband light with wavelengths of 405 nm, 520 nm and 638 nm, transferred to the rigid and fiber optics via a 4.8-mm wide fiber optic cable with ~ 2200 lumens at the cable output in the high-speed mode. The diameter of the fiber optic cable was tailored for the rigid optics. This factor can be responsible for the loss of about half of the light energy when the cable was used with the flexible endoscope.

The spectral characteristics of the illuminator were matched to the sensitivity characteristics of the camera sensor and to haemoglobin absorption. Such a matching allows for appropriate presentation of glottal area tissues with adequate visualisation of blood vessels (with green and violet light: 520 nm and 405 nm correspondingly) and edges of vocal folds (with red light: 638 nm). Laser light source, due to much higher light beam control than in xenon, ensured no excessive heat generation, which was an important parameter for fiber optics. Proper focusing of the camera was set semi-automatically during the clinical examination. The camera system was synchronized with an audio recording module allowing for a time-stamped registration of images of the vocal folds during phonation and the generated air-pressure changes. The acoustic wave signal was sampled at a frequency of 22,050 Hz. The camera collected approx. 200 images during a single recording in quick HSVP mode or 2000 images during normal HSVP mode. For a framerate of 2400 images per second the duration of the video recordings were 83 ms or 830 ms correspondingly. Such a framerate corresponds to a 0.4 ms interval between consecutive image captures. Hence, about 9 audio samples were recorded during this interval. To ensure proper synchronization of video and audio data, additional 1/25 s buffers for storing the audio signal samples occurring before and after video data capture were applied.

The processing pipeline of the image registered with the use of a rigid endoscope consisted of the following steps: removing fixed pattern noise for the camera sensor, debayering, white balance correction, histogram equalization and automatic gamma correction. On the other hand, in the processing pipeline of the image registered by a flexible endoscope after the debayering stage, moiré patterns were removed by a spectral filter that cancelled peaks in the frequency domain.

### Creating a glottal width waveform (GWW) for vocal fold vibration analysis

After software-based optic stabilisation of the image data the kymographic section plots were generated. The HSV can be used not only to assess functional dysphonia and minor organic lesions of glottis but also to evaluate large organic changes of the glottis. For this reason, for parametric analysis of the periodicity of phonation, it is necessary to generate a waveform representing the movements of the vocal folds. Usually, the glottal area waveform (GAW) is applied for this purpose, however, it describes the existing phenomena in a highly averaged way, which means that some nuances of vocal fold oscillations may go unnoticed.

To enable a more accurate periodicity analysis of the oscillation phenomena, for instance the organic pathologies, a waveform was built that reflects instantaneous changes in the glottal area at different glottal lengths levels. By these means we can visualise glottal dynamics. Such a representation is referred to in literature as the glottal width waveform (GWW)^10,19^. To build such a waveform, an appropriate kymographic cross-section was first generated. Then, for the generated cross-section, the region was determined, of a width that is at least twice as large as the width of the glottal area containing the central axis of the glottis. Subsequently, a brightness profile containing the selected area was constructed for each section. Only the red colour component of the image was used to create such a profile, because due to the narrowband nature of the used light source, the brightness profile is practically completely devoid of artifacts related to visible blood vessels. The profile is limited from the top by the values of its weighted average, where the weights were determined proportionally to the darkness of the pixels. The modified profile was iteratively approximated by single periods of sinusoidal functions of decreasing widths. Each new sinusoidal function was positioned centrally relative to the previous minimum. Half the width of the best fit curve was taken as the glottal area width. As a result, it was possible to reliably determine the width of the glottal area in a fully automatic manner and the calculation was based on the shape of the whole glottic area profile rather than on a simple image thresholding method.

### Glottal width waveform (GWW) assessment for parametrization of glottal vibratory cycle characteristics

**Figure 2:**
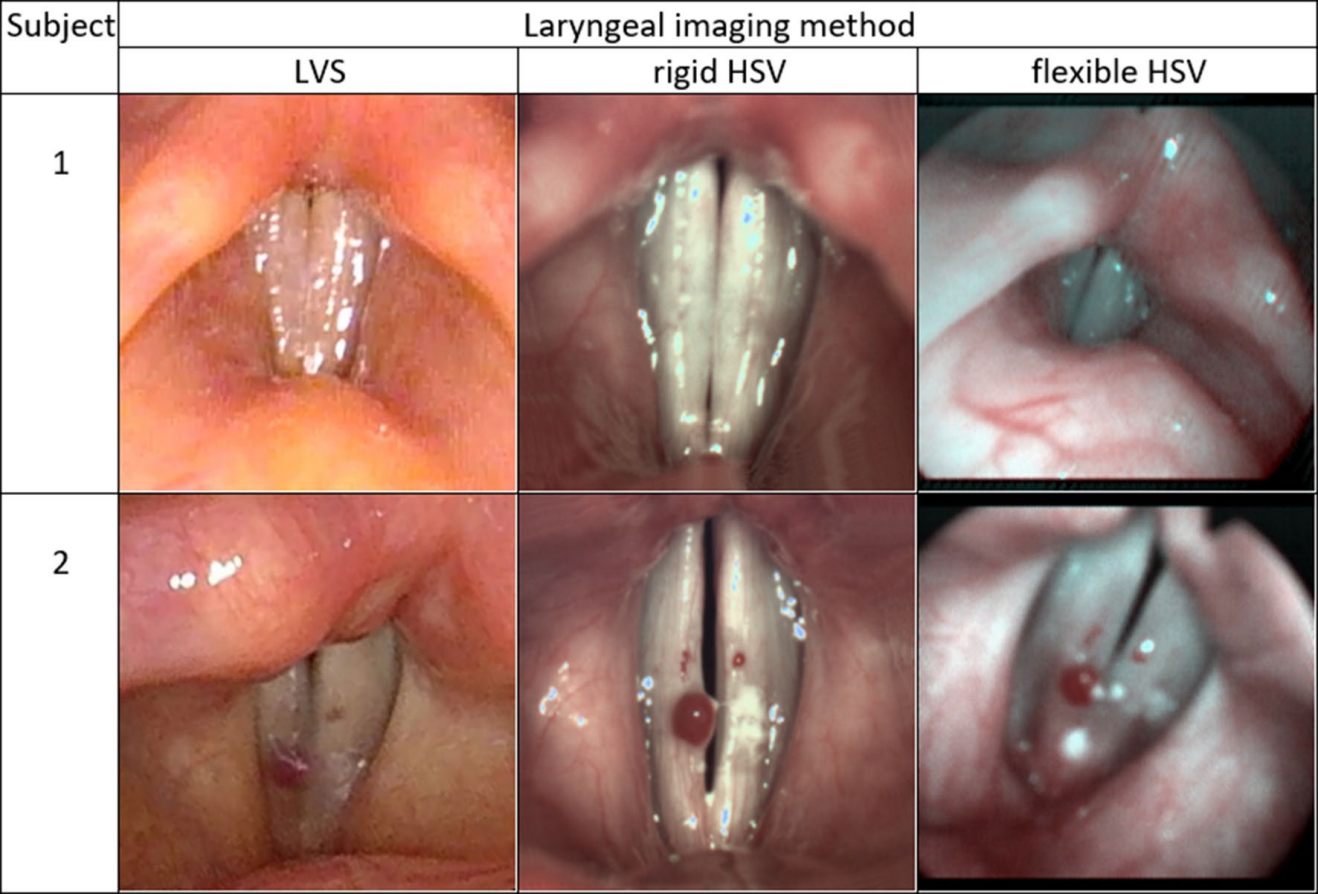
Comparison of glottal images for Subject 1 and Subject 2, obtained using three different methods. From left to right: Laryngovideostroboscopy (LVS); High Speed Videoendoscopy by means of rigid optics (rigid HSV); High Speed Videoendoscopy by means of flexible optics (flexible HSV).

GWW selection for analysis of the phonatory vibration allows, among others, for simpler analysis in a case where an organic lesion divides the glottis area into parts or where there is a large phase difference between the movements of the front and back sections of the vocal folds. When there were no other indications related to the presence of organic changes, the cross-section representing the maximum amplitude of glottal width changes with the full vocal folds closing phase clearly visible were chosen (Fig. 2).

**Figure 3:**
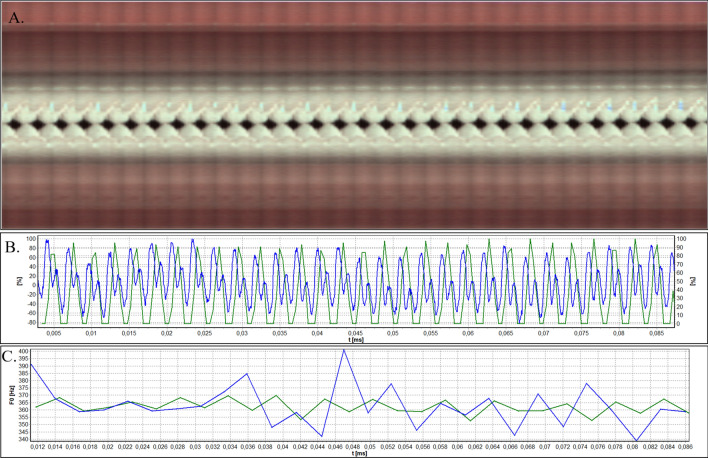
Analysis of HSV data acquired by means of rigid endoscope for Subject 1. (**A**). Videokymogram; (**B**) Comparison of the glottal width waveform obtained from a videokymogram (green line) and the audio signal oscillogram (blue line); (**C**) Comparison of F0 obtained from a videokymogram (green line) and acoustic voice analysis (blue line).

**Figure 4:**
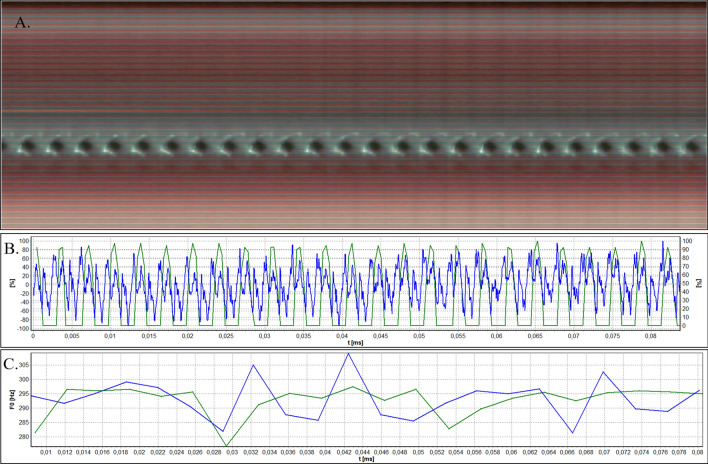
Analysis of HSV data acquired by means of flexible endoscope for Subject 1. (**A**) Videokymogram; (**B**) Comparison of the glottal width waveform obtained from a videokymogram (green line) and the audio signal oscillogram (blue line); (**C**) Comparison of F0 obtained from a videokymogram (green line) and acoustic voice analysis (blue line).

**Figure 5:**
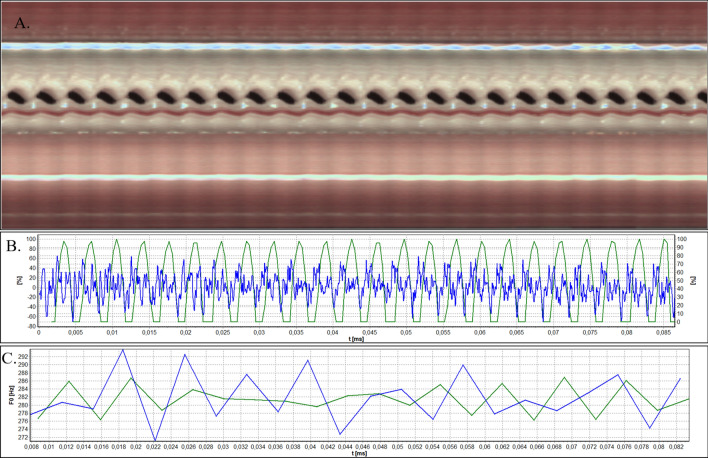
Analysis of HSV data acquired by means of rigid endoscope for Subject 2. (**A**). Videokymogram; (**B**) Comparison of the glottal width waveform obtained from a videokymogram (green line) and the audio signal oscillogram (blue line); (**C**) Comparison of F0 obtained from a videokymogram (green line) and acoustic voice analysis (blue line).

**Figure 6:**
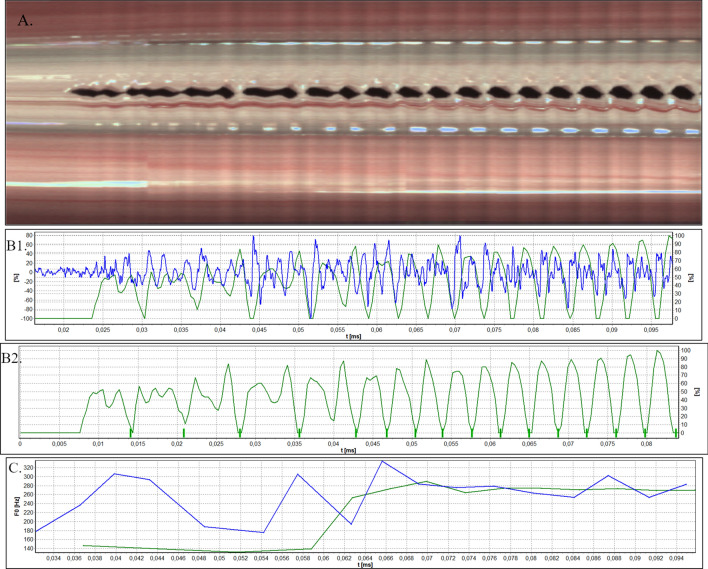
Analysis of HSV data of phonatory onset acquired by means of rigid endoscope for Subject 2. (**A**) Videokymogram; (**B1**) Comparison of the glottal width waveform obtained from a videokymogram (green line) and the audio signal oscillogram (blue line); (**B2**) Glottal width waveform with marked closures of glottis; (**C**) Comparison of F0 for obtained from a videokymogram (green line) and acoustic voice analysis (blue line).

**Figure 7:**
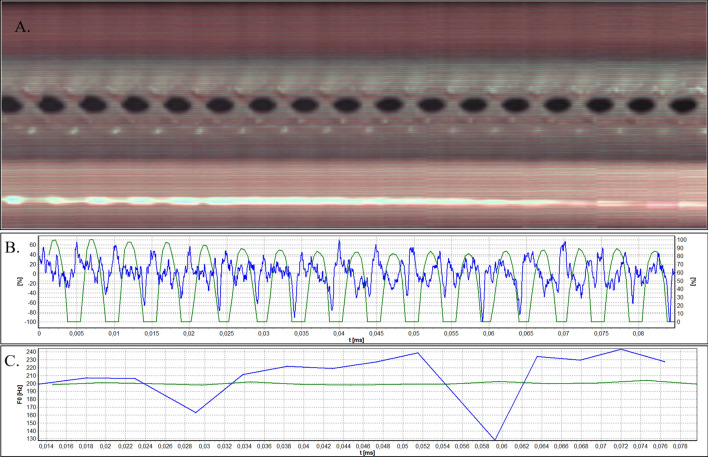
Analysis of HSV data acquired by means of flexible endoscope for Subject 2. (**A**) Videokymogram; (**B**) Comparison of the glottal width waveform obtained from a videokymogram (green line) and the audio signal oscillogram (blue line); (**C**) Comparison of F0 obtained from a videokymogram (green line) and acoustic voice analysis (blue line).

Figures 3B, 4, 5, 6, and 7B show glottal width waveform obtained from a videokymogram (green line) in comparison to the audio signal oscillogram (blue line), corresponding to an audio signal recorded simultaneously with HSV recordings. In order to assess the extent of glottal closure, the values in the glottal width waveform plots represent the momentary opening phase of glottal cycle with respect to the maximum opening (open phase of glottal cycle) in the analysed image sample and are expressed as a percentage. Analogously, sound pressure in oscillograms is expressed as the ratio of the momentary sound pressure to the maximum sound pressure in the given audio samples. This does not pose any limitation for the functional acoustic analysis and allows to avoid the influence of a number of factors disturbing the absolute measurement of sound.

Period and amplitude perturbation measures described below were defined both for the GWW and acoustical signals.

### Period perturbation measures

Perturbation in the period length between different oscillation cycles can be measured by a number of different parameters. The fundamental frequency of the phonatory vibrations that is required to calculate these parameters was identified by the DFT (Discrete Fourier Transform) method as the inverse of the difference in distance between minima of the adjacent periods determined by the Fourier Curve Fitting method. Period perturbation measures were determined for the chart of the glottic width using the following mathematical formulas and Glottal Analysis Tool 2018, developed in Erlangen, Germany^38^.

Mean period of vocal fold oscillations:

$$\overline{T} = \frac{{\mathop \sum \nolimits_{i = 0}^{N - 1} T\left( i \right)}}{N}$$

where *N* is the number of analysed periods.

Mean Jitter value which is a measure of irregularity of the oscillation periods:

$$Jita = \frac{{\mathop \sum \nolimits_{i = 1}^{N - 1} \left| {T\left( i \right) - T\left( {i - 1} \right)} \right|}}{N - 1}$$

Mean Jitter value given in %:

$$Jitt = \frac{Jita}{{\overline{T}}} \times 100 \left[ \% \right]$$

Period Perturbation Factor:

$$PPF = \frac{{\mathop \sum \nolimits_{i = 1}^{N - 1} \left| {\frac{{T\left( i \right) - T\left( {i - 1} \right)}}{{\left( {\frac{{T\left( i \right) + T\left( {i - 1} \right)}}{2}} \right)}}} \right|}}{N - 1} \times 100 \left[ \% \right]$$

Period Perturbation Quotient with *k* averaging area width:

$$PPQ_{k} = \frac{{\mathop \sum \nolimits_{i = k - 1}^{N - 1} \left| {1 - \frac{{k \times T\left( {i - \frac{k - 1}{2}} \right)}}{{\mathop \sum \nolimits_{j = 0}^{k - 1} T\left( {i + j} \right)}}} \right|}}{{N - \left( {k - 1} \right)}} \times 100 \left[ \% \right]$$

where *k* is the width of the local averaging area, it is an odd number;

Period Relative Average Perturbation:

$$\left( P \right)RAP = \frac{{\frac{1}{N - 2} \times \mathop \sum \nolimits_{i = 2}^{N - 1} \left| {\frac{{T\left( {i - 2} \right) + T\left( {i - 1} \right) + T\left( i \right)}}{3} - T\left( {i - 1} \right)} \right|}}{{\overline{T}}} \times 100 \left[ \% \right]$$

### Amplitude perturbation measures

Consecutive amplitudes of the GWW were used to determine the shimmer parameters. The amplitude determined in this way depends both on the amplitude of changes in the glottis width and the changes in the open quotient. As a result, the determined shimmer parameters characterise the changes between the fundamental periods in a more comprehensive way. The following parameters were calculated using the mathematical formulas listed below:

Mean amplitude:

$$\overline{a} = \frac{{\mathop \sum \nolimits_{i = 0}^{N - 1} a\left( i \right)}}{N}$$

Shimmer:

$$Shimmer = \frac{{\frac{1}{N - 1}\mathop \sum \nolimits_{i = 1}^{N - 1} \left| {a\left( i \right) - a\left( {i - 1} \right)} \right|}}{{\overline{a}}} \times 100 \left[ \% \right]$$

Amplitude Perturbation Factor:

$$APF = \frac{{\mathop \sum \nolimits_{i = 1}^{N - 1} \left| {\frac{{a\left( i \right) - a\left( {i - 1} \right)}}{{\left( {\frac{{a\left( i \right) + a\left( {i - 1} \right)}}{2}} \right)}}} \right|}}{N - 1} \times 100 \left[ \% \right]$$

Amplitude Perturbation Quotient with *k* averaging area width:

$$APQ_{k} = \frac{{\mathop \sum \nolimits_{i = k - 1}^{N - 1} \left| {1 - \frac{{k*a\left( {i - \frac{k - 1}{2}} \right)}}{{\mathop \sum \nolimits_{j = 0}^{k - 1} a\left( {i + j} \right)}}} \right|}}{{N - \left( {k - 1} \right)}} \times 100 \left[ \% \right]$$

Amplitude Relative Average Perturbation:

$$\left( A \right)RAP = \frac{{\frac{1}{N - 2}*\mathop \sum \nolimits_{i = 2}^{N - 1} \left| {\frac{{a\left( {i - 2} \right) + a\left( {i - 1} \right) + a\left( i \right)}}{3} - a\left( {i - 1} \right)} \right|}}{{\overline{a}}} \times 100 \left[ \% \right]$$

### Ethical approval

Approval for this study was granted by the Ethical Committee of the Medical University of Lodz (decision no. RNN/96/20/KE 08/04/2020).

## Results

The initial study group comprised 15 subjects. Two females were selected for the extensive analysis of the obtained rigid HSV and flexible HSV results: a normophonic case (Subject 1) and an organic disorder case (Subject 2). Subject 1 was a 23-year old student, non-smoker, reporting no previous voice disorders (VHI- 0- points, G0R0B0A1S0, MPT- 12 s). No abnormal ratings of stroboscopic parameters were observed in her LVS examination. Subject 2 was a 55-year old female, non-smoker, who reported occasional hoarseness and voice breaks (VHI: 30 points, G1R2B1A0S0, MPT: 10 s). Laryngovideostroboscopy revealed haemorrhagic polyp of the right vocal fold located on its upper surface near the edge in the middle one-third. Temporary aperiodicity of phonatory vibrations with incomplete glottal closure was observed.

Figures 3, 4, 5, 6, and 7A present videokymograms obtained from high-speed imaging. On the basis of videokymograms it was possible to conduct the precise detection of the duration of fundamental periods.

Figures 3, 4, 5, 6, and 7B illustrate the graphs of glottal width waveform built from the videokymogram and oscillograms derived from the simultaneously recorded audio samples. The graph of the glottal width waveform GWW (green color) which is a plot of time variations/changes of the instantaneous values of the glottal width allows the examination of kinematics of the vibrating vocal folds in comparison to the oscillogram (blue color) corresponding to an audio signal.

Figures 3, 4, 5, 6, and 7C show graphs of F0 obtained on the basis of videokymograms of high-speed video signals in comparison to those derived from simultaneously recorded audio signals.

GWW and acoustic data enable calculation of period and amplitude perturbation parameters describing vocal fold oscillation presented in detail in Methods (Table 1).

**Table 1:** Parameters obtained from rigid and flexible optics during video and audio HSV examination.

Parameter	Subject 1				Subject 2			
	Video		Audio		Video		Audio	
	Rigid	Flexible	Rigid	Flexible	Rigid	Flexible	Rigid	Flexible
Periods	28	22	28	22	22	14	22	14
F0 Avg (Hz)	361,4	292,1	362,9	293,0	280,8	199,6	282,0	210,9
Jita (ms)	0,06	0,06	0,14	0,11	0,07	0,06	0,13	0,79
Jitt (%)	2,30	1,71	5,01	3,16	2,08	1,13	3,60	16,18
PPF (%)	2,30	1,69	5,02	3,16	2,08	1,13	3,61	13,89
PPQ3 (%)	1,46	0,87	3,05	1,85	1,26	0,56	2,34	10,05
PPQ5 (%)	1,03	0,99	2,70	2,23	0,82	0,57	1,75	14,12
(P)RAP (%)	1,46	0,88	3,06	1,85	1,26	0,56	2,33	11,00
Shimmer (%)	12,15	3,88	13,05	11,78	3,82	2,04	25,50	30,21
APF (%)	12,38	3,87	13,25	11,73	3,79	2,02	25,52	33,67
APQ3 (%)	7,55	2,38	7,10	7,05	2,33	0,92	16,03	12,12
APQ5 (%)	5,92	2,91	7,86	6,91	1,63	0,89	10,60	22,89
(A)RAP (%)	7,39	2,37	6,97	7,12	2,34	0,91	16,13	10,84

Measures are derived from glottovibrograms, two-dimensional spatiotemporal representations of changes of the glottal area during phonation, extracted from HSV recordings. Similar quantitative analysis was performed for acoustic signal, recorded during video examination with a microphone attached to endoscope.

F0 Avg—average fundamental frequency; PPF—Period Perturbation Factor; PPQ3—Period Perturbation Quotient; PPQ5—Period Perturbation Quotient; (P)RAP—Period Relative Average Perturbation; APF—Amplitude Perturbation Factor; APQ3—Amplitude Perturbation Quotient; APQ5—Amplitude Perturbation Quotient; (A)RAP—Amplitude Relative Average Perturbation; where 3 or 5(k) is the odd number of width of the local averaging area.

### Analysis of image (video) and sound (audio) data simultaneously acquired from rigid and flexible high-speed videoendoscopy in Subject 1

In Subject 1 the HSV recordings reveal periodically observed hourglass-shaped glottal closure with minimal anteroposterior glottal compression (Fig. 2). However, kymograms obtained with the use of both rigid (Fig. 3A) and flexible endoscopes (Fig. 4A) indicate regular periodical vocal fold vibrations.

A difference in duration of the minima of the glottis width indicating closed phase (difference in ms) which was revealed by the GWW can be indicative of periodically appearing hourglass-shaped glottal closure seen in HSV evaluation (Fig. 3B). However, more disturbances are visible in the oscillogram which reflects voice production in the vocal tract. The minimal disturbances in the oscillations of vocal folds (Fig. 3B) may have resulted in changes in the acoustic waveform. Similar observation was made for the fHSV video and audio results (Fig. 4B).

From the DFT analysis of an audio signal more instability of F0 is noticeable in comparison to video data (Figs. 3C, 4C), however both average F0 measurements give similar values separately assessed for rigid (F0 video vs audio; 361,4 Hz vs 362,9 Hz) and flexible endoscope (F0 video vs audio; 291,1 Hz vs 293,0 Hz) (Table1). The values of F0 calculated on the basis of rigid HSV recording are much higher than those obtained by flexible optics, which is observed both in video (F0 rigid vs flexible: 361,4 Hz vs 292,1 Hz) and audio recordings (F0 rigid vs flexible: 362,9 Hz vs 293,0 Hz) (Table 1). Higher value of F0 obtained by rigid in comparison to flexible endoscope can be explained by forced phonation. Additionaly, the increase in F0 observed in all measurements could be attributed to a tendency to hyperphonation.

Moreover, most period and amplitude perturbation parameters calculated on the basis of flexible HSV data assume lower values than these quantified on the basis of rigid HSV recordings (Table 1). Again, it can be explained by forced phonation during laryngeal examination by means of a rigid laryngoscope in comparison to more physiological voice production during flexible HSV examination.

### Analysis of image (video) and sound (audio) data simultaneously acquired from rigid and flexible high-speed videoendoscopy in Subject 2

In Subject 2 the tracking of HSV recordings by means of both: rigid and flexible optics revealed hemorrhagic polyp (Fig. 2) seen as the red line in the bottom part of the videokymogram corresponding to the right vocal fold (Figs. 5A, 6A, 7A).

Despite the mass located on the upper surface of the right vocal fold, the oscillations of vocal folds are almost regular. The GWW graph (Figs. 5B, 7B) supports this observation, representing regularly changing glottal width within a glottal cycle during steady phonation. More disturbances are visible in the acoustic waveform (Figs. 5B, 7B) and in F0 curve derived from audio signals (Figs. 5C, 7C). This fact is reflected by higher values of period and amplitude perturbation parameters calculated on the basis of audio in comparison to video signal (Table 1). In particular amplitude perturbation parameters (shimmer group measures) derived from audio signals assume considerably higher values, which indicates changes in stability of the voice. This fact can be explained by the influence of formants and noise from supraglottic part of the vocal tract.

The GWW graphs obtained by means of rigid and flexible laryngoscope do not differ considerably.

In Subject 2, accurate tracing of each vocal fold vibration cycle by means of HSV also enables capturing serious disturbances during onset, that is initiation of vocal fold vibrations. Analysis of image and sound data derived for the onset indicates periodicity disturbances presented in Fig. 6.

The irregularity of vocal fold osscilation (overcycles) is visible in the initial part of the videokymogram (Fig. 6A) as well as in glottal width waveform with distinction of the closed phase (Fig. 6B1, B2). In all recorded onset samples, the closed phase in the glottal cycle occurs every 2–3 periods. On the basis of the glottal width waveform, the exact form of the disturbances might be also reliably determined. This observation is further reflected in the graph of F0 derived from HSV imaging of onset: in the beginning of the graph F0 assumes a lower value (140 Hz) than in the latter part of the graph (260–280 Hz) (Fig. 6C). Analysis of period and amplitude perturbation parameters of vocal fold vibrations derived from high-speed video and audio onset patterns revealed higher values of these measures in comparison to those derived from steady state phonation patterns. For the image data obtained using the rigid endoscope (onset pattern vs steady state phonation), an example comparison is given for the following measurements: Jitter: 9.56 vs 2.08%; Jita: 0.45 vs 0.07 ms; PPF: 8.67 vs 2.08%; Shimmer 11.29 vs 3.82%; APF: 12.46 vs 3.79%. For the audio data derived from onset sample these measurements assume higher values as well: Jitter: 21.44 vs 3.60%, Jita: 0.84 vs 0.13 ms, PPF: 20.57 vs 3.61%, Shimmer 72.58 vs 25.50%, APF: 74.30 vs 25.52%. Similar data were obtained using the flexible endoscope, which further confirmed major disturbances detected in onset in Subject 2.

The comparison of the graphs of F0 obtained from the videokymogram and the acoustic signal for both rigid and flexible HSV recordings (Figs. 4C, 7C) indicates more instability of F0 derived from acoustic data (blue line). Both average F0 measurements give similar values separately assessed for rigid (F0 video vs audio; 280,8 Hz vs 282,0 Hz) and flexible endoscope (F0 video vs audio; 199,6 Hz vs 210,9 Hz) (Table1). The values of F0 calculated on the basis of rigid HSV recording are much higher than those obtained by flexible optics, which is observed both in video (F0 rigid vs flexible: 280,8 Hz vs 199,6 Hz) and audio recordings (F0 rigid vs flexible: 282,0 Hz vs 210,9 Hz) (Table 1).

Most parameters quantifying vocal fold oscillations calculated on the basis of HSV imaging and audio data acquired by means of flexible endoscopy for Subject 2 assume lower values in comparison to these based on rigid endoscopy, similarly as in Subject 1(Table1).

The lowest values of parameters from the shimmer group were obtained by means of fHSV in both subjects. The likely explanation for this fact is forced phonation during laryngeal examination by means of a rigid laryngoscope, but it is not the only reason for this observation. Additionally, acoustic parameters obtained by both rigid and flexible endoscopes were also much higher than image-based parameters. This applies especially to amplitude perturbation parameters which are particularly sensitive to the noise disturbances in supra-glottal part of the vocal tract.

Furthermore, the observed differences between audio- and video-based parameters might have resulted from the technical aspects of the conducted examination. To ensure a smooth test run, the position of the microphone at the camera was not changed when switching from rigid to flexible laryngoscope. In the case of rigid optics, the position of the microphone at the camera guaranteed the minimum required audio data quality, while in the case of flexible optics, adjusting the camera resulted in changing the position and direction of the microphone and could considerably impair the quality of the recorded audio data. This significantly lowered the signal-to-noise ratio, which, in the case of Subject 2 can explain the increased values of most period perturbation parameters calculated on the basis of audio data acquired from flexible HSV recordings in comparison to these acquired from rigid HSV recordings (Table1).

To sum up, in both presented Subjects, comparison of parameters quantifying vocal fold oscillations acquired from rigid and flexible HSV recordings indicate more naturalistic voicing during the examination by means of flexible laryngoscopy.

The HSV recordings by means of fiber-optic laryngoscope have enabled obtaining consistently bright, color high-speed images for both presented subjects (Fig. 2). Even though the HSV images obtained during flexible laryngoscopy are not as satisfying as those obtained from the recordings with the use of a rigid endoscope, they still made it possible to perform reliable quantitative analysis.

## Discussion

This is the first report of successful application of laryngeal flexible High Speed Video with laser light source with simultaneous recordings of the voice sound. Analysis results of image and audio data recorded by a flexible laryngoscope are compared to the results obtained by means of a rigid endoscope. This pilot study explores the potential benefits of using quantitative analysis of image and sound data simultaneously acquired from high-speed laryngovideoendoscopy to improve clinical assessment of phonatory function.

The character of the study is experimental, however seminal, and it presents the results of two female subjects selected from the examined group. In laryngovideostroboscopic (LVS) examination of Subject 1 no deviations were observed in terms of phonatory vibrations. However, HSV examination revealed discrete functional changes which were confirmed in the values of quantitative parameters assessing phonatory vibrations (Table 1). The examination of this subject has revealed a number of shortcomings of videostroboscopy, which is in agreement with other studies^5,15,39,40^. First, the images from LVS are illusory, as they are collected from different glottal cycles, making it cumbersome to investigate aperiodic dysphonic as well as normophonic voices^40,41^ or vibrations during short sustained phonation as in the described Subject 1. For this reason the HSV is currently becoming the tool of great interest and increased introduction into the clinical practice^11,18,31,42,43^. Given that HSV can capture the real-time image of vocal fold vibrations, the HSV imaging presents the true intra-cycle vibratory characteristics of the vocal folds independent of cycle-to-cycle periodicity.

Moreover, accurate tracing of each vibratory cycle by means of HSV enables the visualization of phonatory gestures such as voice onset and offset^36,44^. Additionally, HSV allows for a clear visualization of the initial movement of the vocal folds from resting position to phonatory vibration. Analysis of this transient phase of vocal fold vibration may provide new, valuable information in the assessment of laryngeal function. In the presented study this is seen in the example of overcycles observed in the onset (all samples) for Subject 2 with a diagnosed hemorrhagic vocal fold polyp (Fig. 4). The polypoid lesion is the underlying cause of the phenomenon of overcycles. For certain positions of the vocal folds the lesion partially divides the glottis into two sections, creating separate sources of vibrations from the whole as well as from the upper and lower parts of the vocal folds. This, in turn, results in superposition of several vibrations. On the basis of the kymographic plot and the extracted GWW graph in onset, the nature of a voice disorder can be established in a reliable way. The present observation reveals that voice initiation may reflect the structural integrity of laryngeal system and confirms that investigation of the onset may prove helpful in better elucidating the effects of disease processes on vocal fold vibration^17,44^.

Most laryngeal HSV examinations are usually performed with a rigid transoral laryngoscope^3,13,21,30,45^. Because of the requirement for greater illumination at higher frame rates the HSV imaging systems rely mostly on a camera attached to a 70- or 90-degree rigid laryngoscope. During the examination conducted in this manner the tongue is out and the mouth is in an open position. As the examination relies on forced phonation, the observation of natural phonation function is compromised^35^. Moreover, the examination by means of a rigid laryngoscope can be impossible to conduct, or considerably limited by a patient’s overactive gag reflex. Several prior publications have reported that high-speed video imaging of the larynx through the fiberoptic laryngoscope creates the most favourable conditions for observation physiological manner of voicing, as most of the laryngeal function is retained, including connected speech and singing^36,46–48^. At the same time, as the endoscope is passed through the nose, the sensation of gag reflex is typically reduced, too. All points considered, the flexible HSV proves beneficial both for the clinician and for the examined subject.

One of the most frequently reported disadvantages of flexible HSV imaging is that the obtained colour high-speed images leave much to be desired in terms of image quality. In the pioneering study by Woo and others^35^ in which flexible laryngoscope was used and only black and white HSV imaging was obtained, it was reported that the HSV image can be enhanced by either extra illumination or by image enhancement using digital image noise reduction. As recently underlined in the literature on the subject, the ability to capture colour flexible HSV imaging of the larynx is important for explanation on how organic lesions and tissue irritation affect vocal fold vibratory function^36^. Until now, there have been several studies conducted in the field of providing adequate lighting inside the larynx during flexible HSV^36,45,49^. The US study^36^ suggested that the most effective way of optimizing flexible HSV image brightness was the manipulation of camera gain and adjustment of scope distal tip distance from the glottal plane, whereas the supplemental light fibre bundle provided minimal benefits due to incorrect light diffusion. Adequate lighting becomes doubly relevant in the case of colour imaging.

One of the strongest points of the presented study, conducted in clinical settings, was obtaining colourful flexible HSV images with all their advantages—capability to reflect the structure of glottis and its lesions (e.g. haemorrhagic polyp diagnosed in Subject 2)—without special manipulation with the endoscope tip. This was achieved thanks to the pioneering use of a laser light source. Although, laser light has been used during classical LVS examination^50^ and the HSV examination^49^, its use was limited to pattern projection for triangulation to obtain basic information about 3D glottal geometry.

The authors have not found any reported cases of application of laser light to endoscopic illumination. The present study is therefore, the first study where a laser light was used as the main light source. A better controlled light beam with precisely defined diffusion has made it much more efficient to lead the light into a small lightguide inside of fiber optics, despite the fact that the current version of the Diagnova illuminator (ALIS Lum-MF1) was optimized for the use with rigid optics, which is equipped with a wider fiberoptic cable. In fiberoptic HSV, the imaging is formed through bundles of optical fibers and the limited number of fibers decreases the image resolution. In the system used in this study, advanced spectral algorithms were applied to remove classical moire pattern, but the problem of resolution cannot be simply overcome without using the image sensor chip-on-the-tip solution, which is currently unreachable for high speed imaging. High speed camera sensor has to process and transfer extremely high amount of video data. This requires a large number of parallel ADCs (Analog to Digital Converters) and a significant computing power which results in a large heat generation and large sensor dimensions. To dissipate a large amount of heat without excessive temperature rise, which can be dangerous for the patient, again, a sufficiently large sensor size is required due to the fact that the amount of heat dissipated is proportional to the surface of the object. Short analysis of websites of main video sensors manufacturers (Sony Semiconductor, ON Semiconductor, ams AG, LUXIMA Technology, Pyxalis, STMicroelectronics), suggests that there is currently no trend towards the miniaturization of high speed sensors due to the above mentioned technical problems^36,49^.

Even though the present study shows that the HSV images obtained during flexible laryngoscopy are not as clear as HSV recordings that use a rigid endoscope, it should be underlined that the obtained flexible HSV data enable quantitative analysis of vocal fold vibration in the same manner as in case of HSV data acquired by means of a rigid endoscope. Because of comparative analysis of HSV data acquired from flexible and rigid laryngoscopy, only the voice samples of sustained vowel / i:/ have been taken into account. The presented system can acquire images of the vibrating vocal folds with simultaneous voice sound recording from the subject. The information derived from this combined analysis can be used to compare measures obtained from acousic and image-based analysis for assessment of voice condition in various voice pathologies^7,37,51^. As seen in Table 1, the computed period and amplitude perturbation measures derived from image and sound data simultaneously acquired from high-speed video examination, the values of these parameters differ slightly. Most perturbation measures derived from sound data are larger than those obtained from the image data. These discrepancies may suggest the influence of the noise/ subharmonics from the supraglottal part of vocal tract that is added to the signal generated in the glottis^7,24,28^.

Moreover, the comparison of parameters calculated from the HSV imaging indicates a more naturalistic voicing during the examination conducted by means of a flexible laryngoscope than during examination with the use of a rigid one. These findings are in line with previously conducted studies^35,36,48^. In rigid HSV, the higher value of average fundamental frequency F0 (related to the perceived pitch of voice) and most acoustic parameters may confirm forced phonation in this kind of examination for both described Subjects.

### Limitations of the study

The findings of the presented study need to be interpreted with caution. The laryngeal HSV imaging system was optimized for the use of rigid optics. Therefore, in the first step, modifications will be introduced to increase the brightness of the images obtained from the fiberscope. Moreover, the procedures of data acquisition from the high-speed camera will be expanded with the functions of automatic detection and recording of phonatory gestures and short-term disturbances. Future research will also be undertaken in the subject of estimating the distance of the endoscope tip from the observed structures of the larynx, which will enable a much more accurate assessment of the size of pathological changes.

Automatic voice quality assessment based on the analysis of images from a high-speed camera will also be significantly expanded by introducing automatic measurements for all glottal cross-sections. Methods of automatic determination of other parameters characterizing phonatory vibrations, such as phase characteristic parameters (e.g. Open Quotient) will be developed.

Our preliminary report is limited to a small number of subjects. Future investigation calls for gathering samples of a larger population.

## Conclusions

High-speed video laryngoscopy is a more accurate tool for reliable laryngeal evaluation and assessment of phonatory function than commonly used laryngovideostroboscopy. HSV imaging is not dependent on the assumption of periodicity. Better resolution of the vibratory characteristic and visualization of the cycle-to-cycle differences allows more precise characterisation of the vibratory behaviours in normal and diseased states and the analysis of subtle vocal transients e.g. in phonation onset. The flexible HSV has a capacity to outperform rigid HSV in terms of assessment of the phonatory function in more physiological manner. The present flexible HSV imaging data has enabled qualitative and quantitative assessment of vocal fold vibrations, identifying subtle changes in the voice useful for voice biometrics. Acoustic-based verification approach to HSV images parameterized on the basis of kymographic section and glottal width waveform (GWW) adds substantially to our understanding of the combined effects of glottal source dynamics and the vocal tract transfer function.

The application of laser light source in flexible videoendoscope enables obtaining images of satisfactory quality to assess the nature of glottal organic lesions and its effect on vocal fold vibrations. The presented technology seems promising and potentially beneficial for implementation in clinical practice.

## Data Availability

The data that support the plots within this paper and other findings of this study are available from the corresponding author upon request.

## Abbreviations

(A)RAP: Amplitude Relative Average Perturbation
(P)RAP: Period Relative Average Perturbation
ADCs: Analog to Digital Converters
ALIS: Advanced Larynx Imager System
APF: Amplitude Perturbation Factor
APQ: Amplitude Perturbation Quotient
DFT: Discrete Fourier Transform
ELS: European Laryngological Society
GAW: Glottal Area Waveform
GWW: Glottal Width Waveform
HSV: High-Speed Videoendoscopy
Jita: Mean Jitter value
Jitt: Mean Jitter value given in%
LVS: Aryngovideostroboscopy
MPT: Maximum Phonation Time
PPF: Period Perturbation Factor
PPQ: Period Perturbation Quotient
PQ: Open Quotient
VHI: Voice Handicap Index
